# Plant-Based Vaccines: Antigen Design, Diversity, and Strategies for High Level Production

**DOI:** 10.3390/vaccines10010100

**Published:** 2022-01-10

**Authors:** Elizabeth Monreal-Escalante, Abel Ramos-Vega, Carlos Angulo, Bernardo Bañuelos-Hernández

**Affiliations:** 1 Immunology and Vaccinology Group, Centro de Investigaciones Biológicas del Noroeste, Instituto PoliItécnico Nacional 195, Playa Palo de Santa Rita Sur, La Paz 23096, BCS, Mexico; aramos@pg.cibnor.mx (A.R.-V.); eangulo@cibnor.mx (C.A.); 2 CONACYT—Centro de Investigaciones Biológicas del Noroeste (CIBNOR), Instituto Politécnico Nacional 195, Playa Palo de Santa Rita Sur, La Paz 23096, BCS, Mexico; 3 Escuela de Veterinaria, Universidad De La Salle Bajío, Avenida Universidad 602, Lomas del Campestre, Leon 37150, GTO, Mexico

**Keywords:** viral vectors, influenza, virus-like particles, virus, COVID 19, antigens, biopharming

## Abstract

Vaccines for human use have conventionally been developed by the production of (1) microbial pathogens in eggs or mammalian cells that are then inactivated, or (2) by the production of pathogen proteins in mammalian and insect cells that are purified for vaccine formulation, as well as, more recently, (3) by using RNA or DNA fragments from pathogens. Another approach for recombinant antigen production in the last three decades has been the use of plants as biofactories. Only have few plant-produced vaccines been evaluated in clinical trials to fight against diseases, of which COVID-19 vaccines are the most recent to be FDA approved. In silico tools have accelerated vaccine design, which, combined with transitory antigen expression in plants, has led to the testing of promising prototypes in pre-clinical and clinical trials. Therefore, this review deals with a description of immunoinformatic tools and plant genetic engineering technologies used for antigen design (virus-like particles (VLP), subunit vaccines, VLP chimeras) and the main strategies for high antigen production levels. These key topics for plant-made vaccine development are discussed and perspectives are provided.

## 1. Introduction

Since the initial comprehension of vaccination by Jenner and Pasteur [1], vaccines for human use have been conventionally developed by the production of (1) microbial pathogens or (2) pathogen proteins in mammalian and insect cells, which are then inactivated and/or purified for final formulations, and, very recently, (3) by using RNA or DNA [2]. Another approach for antigen production is the use of plants as biofactories, which was initially proposed approximately three decades ago [3]). In this period of time, the laboratory-assayed vaccines have reached clinical application. At present, influenza and COVID-19 plant-made vaccines have reached Phase 3 clinical trials, and their results are promising to carry them to commercialization. Ward et al. reported the efficacy, immunogenicity, and safety of a plant-derived, quadrivalent, influenza virus-like particle vaccine in adults and older adults in two Phase 3, multicenter, randomized trials. The results showed that the plant-derived vaccine is protective, well tolerated, and that no major safety signals arose in the participants of the clinical studies [4]. For the influenza virus, the importance of new platforms for vaccine production is due to (1) the humanized virus problem (adaptation to human cell receptors), which minimizes the efficacy of egg-produced vaccines; (2) the use of eggs in case of influenza pandemics could be self-defeating because they commonly originate in birds, so hens could be affected and produce less eggs, and (3), the virus could be lethal in chicken embryos and antigen production could be affected [5].

The emergence of severe acute respiratory syndrome coronavirus-2 (SARS-CoV-2) has also demanded different strategies for vaccine production. Firstly, RNA and DNA vectors have been used to develop the widely distributed vaccines from Pfizer, Moderna, AstraZeneca, CanSino, and Sputnik [6]. Plant-made vaccines against COVID-19 disease have been also developed, mainly producing VLP-based vaccines, and two companies are bringing them to clinical approval. Medicago (CAN) and Kentucky BioProcessing Inc. (Owensboro, KY, USA) have proceeded to Phase 3 clinical trials and several other plant vaccines based on protein subunits are in the preclinical phase [7,8].

Other strategies in the preclinical phase have used plant vaccines administered orally in prime-boost immunization regimes. In this strategy, an injectable vaccine is applied as a prime-boost vaccine and an edible one as a booster. The main application of this strategy has been to improve the vaccine against poliovirus [9]. In this study, the plant oral vaccine as booster improved IgG- and IgA-level production in immunized mice. Notably, a new approach in generating an oral vaccine for poliovirus was to produce it in the chloroplast of edible lettuce [10].

Methods for genetic plant transformation have been developed to produce heterologous proteins in plant cells in the last 30 years. Initially, plants were genetically engineered by Agrobacterium-mediated nuclear transformation and later by chloroplast via the biolistic method [11,12,13]. These fundamental methods established the developmental basis for a wide number of transformation procedures that nowadays are applied for different designs of vaccines produced in plants (whole organism, specific tissue, and cell culture) and microalgae. An attractive approach is the design and production of virus-like particles (VLPs) in plants as subunit vaccines. This strategy has been useful to produce plant VLPs to fight against infectious diseases even at the industrial scale [8,14].

On the other hand, antigen selection is a key issue for plant-made vaccines. Experimental antigens—for which protective efficacy has been demonstrated at the preclinical or clinical level—have been selected to be produced in plants as a potential low-cost platform option [15,16]. Additionally, antigens can also be selected by immunoinformatic (in silico) approaches such as reverse vaccinology, using computational servers and software that predicts the potential immunogenicity of a given pathogen protein [17].

This review describes the elemental basis of antigen in silico design, plant transformation methods, recent VLP developments, and the most advanced vaccines produced in plant cells, highlighting the main plants used as vaccine biofactories.

## 2. Genetic Antigen Design for Subunit Vaccine Development

Subunit vaccines (SUV) arise from recombinant DNA and genetic engineering, which provide the opportunity to specifically select one or more immunoprotective antigens and produce them in another organism. Thus, the rapid growth of genomic information in database banks and the possibility of sequencing complete genomes allows the use of bioinformatics tools to quickly explore whether a certain protein pathogen has potential for use as an SUV. These bioinformatic tools can be based on (a) reverse vaccinology (RV), to evaluate the characteristics of multiple antigens of a given genome; (b) immunoinformatics, for the selection of immunogenic peptides from selected antigens; and (c) structural vaccinology, for searching for the best three-dimensional conformations of the vaccine protein (Table 1).

The main RV computational tools available are New Enhanced Reverse Vaccinology Environment (NERVE) [18], Vaxign [19], VaxiJen [20] Jenner-predict [21], and VacSol [22]. RV emerged in 1997 with the first application of this method with Group B meningococcus (MenB) [23]. Currently, RV is based on (1) searching for the cellular location of pathogen proteins (e.g., LOCATE and LocDB [24,25]), where those that are exposed are usually the most selected for their rapid interaction with the cells of the immune system; (2) adhesin properties, where those pathogen components with the highest ones could be considered more immunogenic (e.g., SPAAN software [17]); (3) antigenicity, where the use of VaxiJen was the first RV software with a machine-learning strategy and a non-alignment approach for antigen prediction based on candidate antigen selection according to protein physicochemical properties [20]; (4) similarity, avoiding the use of sequences too similar to the host [26].

Immunoinformatics is applied to select the most probable immunogenic peptides to design multiepitopic vaccines. The software to be used can be directed to searching for (a) a cellular response by the affinity of peptides to the major histocompatibility complex I (MHC I, specific for cytotoxic T lymphocytes (CTL) [27,28]), or (b) a humoral response by affinity to specific B lymphocytes combining with selected peptides with affinity to the major histocompatibility complex type II (MHC II).

MHC I class molecules consist of heavy chains complexed with β2-microglobulin, which bind in the endoplasmic reticulum to peptides processed by the proteasome, and later display them on the cell surface to CTLs. High affinity at peptide-MHC I binding is difficult to dissociate where the amino-terminal of the peptide binds to pocket A, carboxyl-terminal to pocket F, and the preferred length of the peptide is usually 9 amino acids [29] (Table 1).

MHC II molecules consist of transmembrane chain proteins (α and β), expressed in the membrane of antigen-presenting cells (APC), mainly in dendritic cells (DC) [30] to show antigens to lymphocytes T CD4+. These peptides come from degraded proteins in the endosome, making it possible to respond to extracellular antigens [31]. The peptides that bind to MHC II pockets are usually 15 amino acids long and can extend outward from the peptide–MHC II binding site [29] (Table 1).

On the other hand, the most used servers for predicting epitopes for B cells are: BepiPred-2.0 [32], DiscoTope 2.0 Server [33], ABCpred [34], and COBEpro [35] (Table 1).

Structural vaccinology is used once the epitopes have been selected to organize them in the best way for a correct three-dimensional conformation with the best immunogenic properties for conformational and linear epitopes, which implies estimating tertiary structure. Subsequently, this structure must be refined and finally validated using different software (Table 1) to obtain a better quality model.

**Figure 1:**
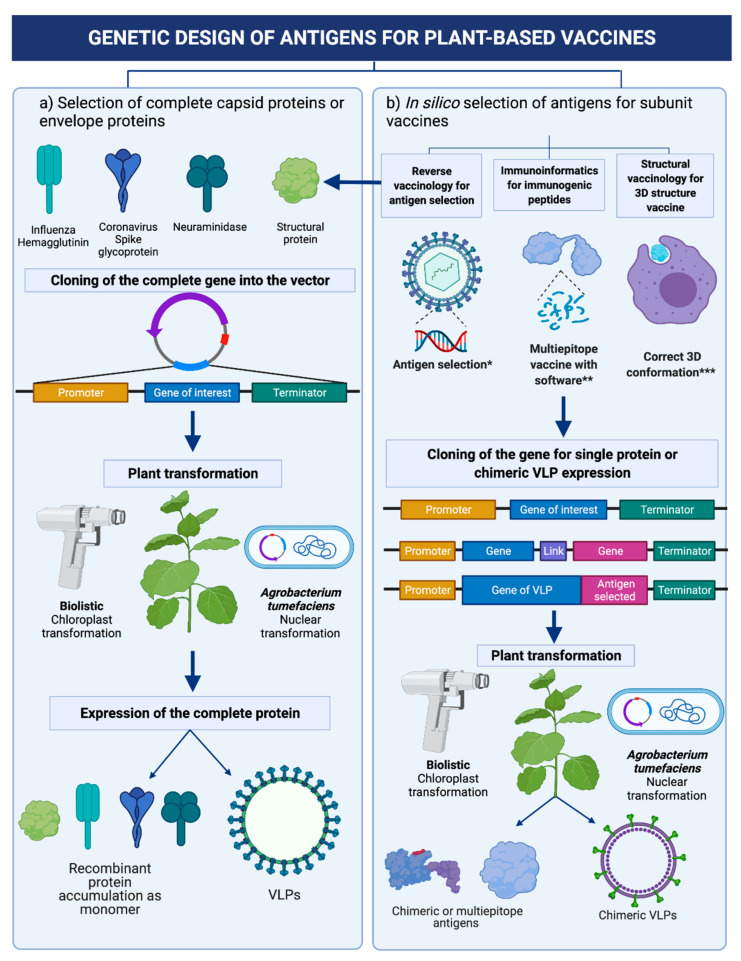
Strategies for designing and producing subunit vaccines using plants as biofactories. (**a**) Expression of complete antigenic viral protein (commonly surface proteins), (**b**) Expression of antigens (fragments of the viral proteins recognized by the immunological system) * Cellular location; adhesin properties; antigenicity; avoid similar host proteins. ** Cellular response by affinity to MHC I or CTL; or humoral response by affinity to MHC II or B lymphocytes. *** Tertiary structure prediction; refinement and validation; molecular docking or dynamics.

A good option to complement the in silico studies for multi-epitope vaccine development is the use of molecular docking to evaluate interaction with a determinate receptor of the immune system of interest. This approach can be explored through the servers PatchDock [36] and Autodock Vina [37]. Molecular dynamics can also be performed with software such as NAMD and VMD [38]. Figure 1 depicts the general route in subunit vaccine design considering the immunoinformatic approach.

## 3. Virus-Like Particles (VLP) and Chimeric Virus-Like Particles (ChVLP) for Subunit Vaccine Production in Plants

Subunit vaccines tend to have low immunogenicity, making the use of adjuvants, higher doses, or booster schedules necessary. Viral virus-like particles (VLPs) are a model of subunit vaccines that are more immunogenic than the three-dimensional formation of individual antigenic proteins. VLPs are a set of repeated structural viral proteins capable of assembling in sizes from 22–150 nm, where 40-nm size has been considered as optimal for recognition by dendritic cells [39]. VLPs have been considered to be a safer option than killed or inactivated pathogen vaccines since they lack genetic material, allowing them to have up to three different protein forming layers from a single structural antigen [40]. In this sense, VLPs can be enveloped or non-enveloped. Non-enveloped VLPs do not include any components of the producer cell and are the most widely used in clinical trials [15]. On the other hand, enveloped VLPs are more complex, including producer cell membrane components with antigens exposed on the outer surface [41].

Since the first VLP produced by Valenzuela et al. [42] consisted of the HBsAg antigen, multiple VLPs have been produced using various platforms. Vaccine production in plants has the advantages of low production costs, high yields, and complex protein production to allow conformation of VLP structures [15,16]). The production of the first antigen in plants (HbsAg) [3] has led to a wide variety of vaccine antigen prototypes of VLP structures produced in plants [3,43]. The most recent are mentioned in Table 2. As noted, the most recent studies of VLPs in plants have used *Nicotiana benthamiana* because of its great capacity to form complex structures with high yields [44,45], although *Nicotiana tabacum* and *Arabidopsis thaliana* have also been used [46], as well as potato, tomato, and lettuce [10,47,48]. Edible plant-made vaccines are mainly interesting when oral administration is used as the inoculation route.

A more recent model of VLPs is focused on the development of chimeric VLPs, which have the advantage of being multivalent, that is, made up of antigens from different pathogens [49,50] and even able to incorporate functional RNA segments [51]. One of the advantages of the chimeric VLP approach is multivalence and the fact that some epitopes or antigens could serve as adjuvants for others. However, some limitations have been identified with the correct folding of the protein, so strategies must be included in the construction design to overcome such problems [52]. Table 2 shows the main antigens recently produced as VLPs.

Another version of chimeric VLP is achieved by inserting immunogenic peptides in the VLP of plant viruses instead of other mammalian viruses. To ensure that the modified capsid protein (CP) of the plant virus shapes the VLP, the added peptide should be located in the surface structure, which is commonly achieved by cloning the antigenic peptide at the N or C terminal of the CP protein [14].

## 4. Plant Genetic Engineering Transformation Methods for Subunit Vaccine Production

### 4.1. Stable Nuclear Transformation

Plant cell nuclear transformation was developed four decades ago and has been mainly achieved by *Agrobacterium tumefaciens* transfection [11,12]. In this method, the DNA containing the transcriptional unit (promoter-gene (antigen)-terminator) for protein expression is cloned in a binary plasmid, commonly pBI121 and pCambia [58]. Then, a plasmid containing the transcriptional unit of the antigen is introduced into *A. tumefaciens*, usually by electroporation. Next, agrobacterium containing plasmids is co-cultured with plant leaf or stem fragments, followed by a complete transgenic plant regeneration through organogenesis or embryogenesis processes [59]. Finally, transgenes and proteins can be detected in fully regenerated plants to confirm genetic transformation and antigen expression.

Initially, the model plant *Nicotiana tabacum* was the main plant used for vaccine production [60]. Currently, many other plants have been considered for vaccine production, some of which have advantages for oral vaccine formulation, such as lettuce and carrots [61,62,63,64]. In agrobacterium-mediated nuclear transformation, plasmids contain a fragment between the T borders (right and left) that is genetically recombined with the nuclear genome upon delivery [65,66]. This fragment harbors a gene that codes for a trait of antibiotic resistance to select transformed cells. Those transformed cells are cultured in media with regulators and hormones for full plant regeneration [67,68]. Gene expression and antigen production in transgenic plants is controlled by promoters that can be constitutive or inducible by a substance or physical conditions, e.g., alcohol, NaCl, temperature, or light [69,70,71]. Remarkably, viral genetic elements can be added into the genetic construction to amplify the number of gene copies [72,73] and protein yields.

### 4.2. Transient Nuclear Transformation

The transient nuclear expression approach is considered when transformed plant cells must be maintained for a short period (two to 10 days). In this case, transgene integration into the nuclear genome is not forced by the antibiotic selection. However, many gene copies are delivered into the nucleus and are capable of producing messenger RNA for high recombinant protein production [74,75]. Transient transformation can be obtained by different strategies where the main aspect is to “infect” a great number of plant cells by using any (or jointly) *Agrobacterium tumefaciens* or viral vectors containing the gene coding antigen [76]. Transient and stable transformation can also be applied to plant cell culture or to microalgae where antigen production could be more uniform and manufacturing practices more efficient, especially in the containment of genetically modified cells [77].

One of the most known viral vectors used in transient expression was developed using elements of the RNA of the *Tobacco mosaic virus* (TMV). Icon genetics named this system magnICON© (DENCA, Tokyo, Japan) and the procedures of plant transformation magnifection [78,79]. Currently, a wide repertory of viral vector types is available, and practically any type of virus could be used to design an expression vector. Recently, the *Bamboo mosaic virus* of the genus potexvirus was used as viral vector to produce Japanese Encephalitis Virus antigens in ^plants [80]. Viral vectors based on plant geminiviruses have been widely used for protein production of biopharmaceutical interest [81,82,83,84]. The mechanism of rolling circle replication allows replicon delivery (copies of transgenes) and can be designed to produce more than one protein in plant cells [85]. Notably, the specific ability of each virus to evade plant immune systems and replicate into the plant cells determines the success of the viral vector in achieving high antigen production.

### 4.3. Chloroplast Transformation

Chloroplast transformation was the first genetic modification of a green cell, performed approximately three decades ago [13]. Initially, the transformation was conducted to create antibiotic-resistant cells [86]. Chloroplast transformation was first achieved through biolistic procedures [87,88], where gold or tungsten microparticles (~10 µm) are coated with genetic material containing minimal gene expression units and then projected into the target cells for transformation [87,88]. Once in the cell, DNA molecules can be integrated into the chloroplast genome through DNA recombination mechanisms [89]. Other plastid organelles have been transformed, such as chromoplasts (plastids in fruits) and those in tubers [90,91,92]. Currently, antigen production in the chloroplast has advantages including higher recombinant protein production than that of stable nuclear transformation, which is determined by the copy number of chloroplast genomes in a given plant [93]. Thus, the chloroplast could be used to produce proteins for subunit vaccines. However, the chloroplast does not have post-translational modification machinery, which is a disadvantage when a post-translational modified protein is necessary for vaccine production.

## 5. Main Plants Used as Biofactories in Vaccine Production

Plants have been used as alternatives for low-cost biopharmaceutical production while reducing the threat for contamination by pathogens and exploiting their great industrial potential. The use of plants for protein recombinant production dates to 1990, when human serum albumin was produced in tobacco. Several plant-based vaccines have been produced against both human and animal pathogens. When vaccines are produced in plants, special attention is focused on evaluating the possibility of the vaccines causing allergies [94]. In this regard, each plant-made vaccine, either purified or in whole-raw material, should be evaluated for security as one of the first steps of vaccine development. For instance, Ward et al. demonstrated that plant-made VLPs influenza vaccine had no allergic or hypersensitivity reactions in subjects during Phase I/II clinical trials [95].

Plant species or specific tissues in a particular plant for commercial recombinant protein production have considerable variability. Several plants, including potato, corn, rice, and soybean, among others, have been used to produce recombinant proteins, including the Hepatitis B vaccine produced in lettuce [96]. However, tobacco and alfalfa have been commonly used because of their high biomass and seed yield and short life cycle.

Seeds can be attractive because of their stability in long storage periods compared to other plant materials. For instance, the companies SemBioSys Genetics, and Ventria Biosciences have seed-based recombinant production systems [97]. Proteins of different molecular weights have been expressed in plant seeds [98], and attempts have been made to develop vaccines in seeds. For example, the *Hepatitis B virus* (HBV) surface antigen (HBsAg) SS1 was produced in rice using the seed-specific Glub-4 promoter. Moreover, mice immunized with this vaccine prototype produced specific antibodies against both S and preS1 epitopes, demonstrating the potential of rice-derived SS1 antigen as an alternative HBV vaccine for Hepatitis B disease [99].

Transgenic potatoes also have been demonstrated to be an excellent expression system. Among the antigens produced in potatoes, the enterotoxigenic *Escherichia coli* (ETEC) labile toxin B-subunit (LTB) [100], and the Norwalk virus capsid protein (NVCP) [101] were promising candidates in clinical trials. Remarkably, the administration of corn seed-made LTB in clinical trials demonstrated similar outcomes to those of the potato study [102]. Another interesting crop used for vaccine production are tomatoes, which have been genetically modified to produce a rabies vaccine [103].

An important aspect when producing vaccines in plants is the containment of transgenes. In this arena, Murphy [104] has reviewed diverse strategies for biocontainment of transgenes, of which the main ones are using male sterility in transgenic plants, transplastomic plants, inducible and transient expression systems, and plant cell cultures instead of whole plants.

A key topic of plant-based vaccines is the possibility of direct oral delivery. In this aspect, when the vaccine is ingested orally, the antigens are expected to be protected from acids and enzymes in the stomach via bio-encapsulation because human digestive enzymes are incapable of breaking down glycosidic bonds in carbohydrates that make up the plant cell wall. However, when intact plant cells containing the vaccine reach the small intestine, commensal microbes digest the cell wall releasing the antigens. When antigens are fused with suitable transmucosal adjuvants (e.g., cholera toxin non-toxic subunit B (CTB)), they are delivered more efficiently to the immune or circulatory system [105,106].

The edible crops mostly used for generating plant-based vaccines have several advantages when compared with the same products made with other plant species. For example, in tobacco (*Nicotiana tabacum* or *N. benthamiana*) plants, the antigen needs to be subsequently purified before being tested, and this process can represent almost 80% of the total vaccine production cost [107].

Moreover, if the vaccine is maintained in lyophilized conditions, cold chain facilities would not be needed to stock and deliver the respective plant material, meaning greater cost efficiency compared to conventional mammalian or fermentation-based vaccines.

## 6. Plant Vaccines Today and Perspectives

Recently, Ward et al. [4] described Phase 3, (NCT03321968) of a quadrivalent, recombinant, virus-like particle (VLP) influenza vaccine produced in plants. This platform is based on transient protein expression in *N. benthamiana* and yields VLPs bearing hemagglutinin (HA) protein trimers that are combined in a quadrivalent vaccine (QVLP). These authors showed 96.3% efficacy, since a lot-to-lot study was carried out just prior to two pivotal placebo-controlled efficacy trials of the same plant-derived QVLP vaccine in ~23,000 adult subjects ≥ 18 years of age, and supported earlier findings of the safety profile and immunogenicity of the plant-derived QVLP, demonstrating the consistency with which it can be produced [108].

Although several vaccines against COVID-19 have been already delivered, different companies are developing plant-made vaccines [8] and some of them are in process of clinical trials [109,110].

On the other hand, Medicago (Quebec, Canada), a biopharmaceutical company, recently announced an FDA-approved CoVLP plant-derived vaccine against COVID-19 administered alone or with AS03 or CpG1018 adjuvants from the GSK Company (Brentford, Middlesex, UK). In the preliminary study, the co-primary outcomes were short-term tolerability/safety and immunogenicity of CoVLP formulations assessed by neutralizing antibody (NAb) and cellular responses [111]. Secondary outcomes in this study included safety and immunogenicity assessments up to 12 months after vaccination. Especially in COVID vaccines, Kumar et al. have reviewed the main companies that are working on developing plant-made vaccine platforms. Medicago and Kentucky BioProcessing Inc (Owensboro, KY, USA) have proceeded to the most advanced clinical trials [112].

These examples highlight that plant-made vaccine platforms are a reliable commercial option in the 21st century. Research groups around the globe are mainly working on a major challenge: increasing protein yields. Nevertheless, some specific commercial niches are being covered by plants, such as glucocerebrosidase against Gaucher’s disease [113]. Laere et al. [114] questioned why a commercial plant-produced vaccine had not been made after two decades of plant vaccine development. The authors pointed out three particular challenges: (1) selection of antigen and plant expression hosts; (2) dosage consistency; and (3) vaccine manufacturing, according to good manufacturing practice (GMP) procedures. Today, companies like Medicago and Kentucky BioProcessing have overcome these limitations.

## 7. Conclusions

Plant-based vaccination is evolving. Improvements and innovations to plant biotechnology platforms have been made by incorporating immunoinformatic tools, genetic engineering methods, and strategies for high yield recombinant vaccine production. Firstly, most of the immunoinformatic tools are free of charge and offer excellent opportunities for novel vaccine designs. In the near future, artificial intelligence will be the central motor for predicting reliable antigens and epitopes for subunit vaccine design. Secondly, it is envisaged that genetic engineering tools can be improved for transient expression as an approach to increase protein yields, with special focus on organic nanoparticles called VLPs to fight against viral diseases. Thirdly, with the fact that industry is involved in the current pandemic challenge and a plant-made vaccine against COVID-19 is now available, new investment for this biotechnological platform could expand in the following years. Future efforts should be directed to moving the experimental success of other plant-made vaccines to clinical applications.

## Figures and Tables

**Table 1:** Computational sources for vaccine development (accessed on 26 December 2021).

Used for	Bioinformatic Tool	Link
Reverse vaccinology	NERVE	http://www.bio.unipd.it/molbinfo/
	Vaxign	http://www.violinet.org/vaxign/
	VaxiJen	http://www.ddg-pharmfac.net/vaxijen/VaxiJen/VaxiJen.html
	VacSol	https://sourceforge.net/projects/vacsol/
MHC I—CTL Epitope prediction	EpiJen	http://www.ddg-pharmfac.net/epijen/EpiJen/EpiJen.htm
	MHCPred	http://www.ddg-pharmfac.net/mhcpred/MHCPred
	NetMHC 4.0 Server	https://services.healthtech.dtu.dk/service.php?NetMHC-4.0
	NetCTL 1.2 Server	http://www.cbs.dtu.dk/services/NetCTL/
	NetCTLPan-1.1	https://services.healthtech.dtu.dk/service.php?NetCTLpan-1.1
	IEDB Analysis Resource	http://tools.iedb.org/mhci/
MHC II—B Cell Epitope prediction	NetMHCIIpan 4.0 Server	https://services.healthtech.dtu.dk/service.php?NetMHCIIpan-4.0/
	NeonMHC2	neonmhc2.org
	NetMHCII 2.3 Server	http://www.cbs.dtu.dk/services/NetMHCII/
	BepiPred-2.0	https://services.healthtech.dtu.dk/service.php?BepiPred-2.0
	DiscoTope 2.0 Server	https://services.healthtech.dtu.dk/service.php?DiscoTope-2.0
	ABCpred	https://webs.iiitd.edu.in/raghava/abcpred/ABC_submission.html
	COBEpro	http://scratch.proteomics.ics.uci.edu/
MHC I—MHC II Epitope prediction	SYFPEITHI	http://www.syfpeithi.de/bin/MHCServer.dll/EpitopePrediction.htm
	NetMHCpan 4.1	https://services.healthtech.dtu.dk/service.php?NetMHCpan-4.1/
	EpiVax	https://epivax.com
Structural vaccinology	Phyre2 server	http://www.sbg.bio.ic.ac.uk/phyre2/html/page.cgi?id=index
	GalaxyWEB server	http://galaxy.seoklab.org/
	SWISS-MODEL	https://swissmodel.expasy.org
	GalaxyRefine2	http://galaxy.seoklab.org/cgi-bin/submit.cgi?type=REFINE2
	MolProbity	http://molprobity.manchester.ac.uk
	ProSA	https://prosa.services.came.sbg.ac.at/prosa.php
	Saves	https://saves.mbi.ucla.edu/
	PatchDock	https://bioinfo3d.cs.tau.ac.il/PatchDock/php.php
	Autodock Vina	http://vina.scripps.edu/

**Table 2:** Main antigens recently expressed as viral virus-like particles (VLPs).

VLP Antigen/Virus	Plant Host	Transformation Method and Yields	Immunization Scheme	Findings	Reference
D antigen (PV3)/*Poliovirus*	*N. benthamiana*	Transient expression by transformation with *A. tumefaciens* method. Yields: 60 mg/kg of infiltrated plant tissue.	Mice received one or two intraperitoneal injections of 0.5 human doses of purified VLPs and were challenged with poliovirus.	VLPs in one and two doses induced similar levels of neutralizing antibodies and protection against a viral challenge.	[45]
CP/PCV-2	*N. benthamiana*	Transient expression by transformation with *A. tumefaciens* method. Yields: 6.5 mg/kg of leaf wet weight.	Mice received 3 subcutaneous injections of 10–20 μg VLPs.	VLPs induced specific antibodies for PCV-2 at 42 days post-immunization.	[53]
H1, H5/*Influenza virus*	*N. benthamiana*	Transient expression by transformation with *A. tumefaciens* method. Yields: not reported.	Not applied	VLPs mimic the structure and initial virus-APC interaction of influenza virions.	[54]
H1, H5/*Influenza virus*	*N. benthamiana*	Transient expression by transformation with *A. tumefaciens* method. Yields: not reported.	Not applied	VLPs were structurally similar and stable for at least one year at 4 °C, interacted with and activated APCs analogous to influenza virions	[55]
VP2,VP3,VP5,VP7/*African horse sickness virus* (AHSV)	*N. benthamiana*	Transient expression by transformation with *A. tumefaciens* method. Yields: Not reported	Intramuscular immunization of horses with 200 μg or 100 μg total VLPs protein plus Pet Gel A adjuvant	All immunized horses showed specific antibodies after the second dose. However, those that received the highest dose had higher neutralizing titers.	[56]
VP6/(RVs) GI.4,GII.4-2006a/(NoVs)	*N. benthamiana*	Transient expression by transformation with *A. tumefaciens* method. Yields: Not reported	Mice received intradermally 0.3 µg three times or 1 µg of GI.4 and GII.4-2006a VLPs combined with 10 µg of VP6.	VP6 had an adjuvant effect in the production of antibodies against NoV VLPs.	[57]
VP0, VP1, VP3/FMD	*N. benthamiana*	Transformation with *A. tumefaciens* method. Yields: ∼0.030 μg/g of fresh leaf material.	Mice were immunized subcutaneously four times with 5 μg VLPs plus Montanide ISA 50 V 2 (Seppic) adjuvant.	→ The VLPs produced in plants were assembled without the need for the 3C protease precursor, in comparison with those expressed in other platforms, such as mammalian and insect cells. → VLPs were significantly immunogenic in mice.	[44]

Abbreviations; VLP: virus-like particles; APC: antigen-presenting cells; FMD: *Foot-and-mouth disease*; RV: *rotaviruses*; NoVs: *noroviruses;* AHSV: *African horse sickness virus*; H1: hemagglutinin (A/California/07/2009 (H1N1)); H5: hemagglutinin (A/Indonesia/05/2005 (H5N1)); PCV-2: *Porcine circovirus type 2;* CP: capsid protein.

## Data Availability

Not applicable.
